# Vaccination coverage against COVID-19 in a Tunisian general hospital

**DOI:** 10.11604/pamj.2021.40.101.31911

**Published:** 2021-10-14

**Authors:** Hamdi El Kefi, Khira Kefi, Nejla Stambouli, Ridha Belaej, Mohamed Jalel Hmida, Abdelaziz Oumaya

**Affiliations:** 1 Psychiatry Unit, Military Hospital of Instruction of Tunis, Tunis, Tunisia,; 2 Military Hospital of Instruction of Tunis, Tunis, Tunisia,; 3 Preventive Medicine Unit, Military Hospital of Instruction of Tunis, Tunis, Tunisia,; 4 Department of Anaesthesiology and Intensive Care, Military Hospital of Instruction of Tunis, Tunis, Tunisia

**Keywords:** Vaccination, COVID-19, hospital staff

## To the editors of the Pan African Medical Journal

In August 2020, well before the discovery of the COVID-19 vaccine, we conducted a study at the Military Hospital of Tunis on the acceptability of the COVID vaccine. Our study was published in the Pan African Journal of Medicine. Because of the fear of side effects of the vaccine, several training and awareness-raising activities were carried out but did not achieve the desired rate of vaccination coverage. Only compulsory vaccination has made it possible to achieve a vaccination coverage close to 100%.

The year 2020 was marked by the start of the COVID-19 pandemic. Hospital staff have been at the forefront of the fight against this virus. This situation has caused a high level of stress among health care workers, mainly due to the fear of infection, contamination of family members and the death of many patients. The lack of a cure and an effective vaccine were major stressors [1]. In anticipation of the discovery of a COVID-19 vaccine, a study was conducted at the Military Hospital of Tunis between August and September 2020 on the acceptance of vaccination against this virus [2]. The results of the study were published in the Pan African Journal of Medicine.

A total of 398 personnel staff of the Military Hospital were randomly selected to participate in our study, composed of 9% (n=36) physicians, 0.9% (n=3) pharmacists, 41.3% (n=164) paramedics, 16.1% (n=64) cleaning staff and 32.7% (n=131) administrative staff. The rapid discovery of the vaccine was hoped by 97% (n=386). However, only 58% (n=231) agreed to be vaccinated by the COVID-19 vaccine. We concluded that the apprehension about vaccination does not appear to be sparing the future COVID-19 vaccine. Fear of vaccine side effects outweighs fear of the disease, even among hospital staff [2]. At the end of the study, several training sessions on the mode of propagation of the virus and the means of prevention against COVID-19 were conducted by the vaccination committee and the training and awareness subcommittee of the Military Hospital of Tunis. Vaccination awareness activities were planned for the nursing staff to reduce the fear of the vaccine. Several vaccines against COVID-19 have been developed and vaccination campaigns started in December 2020. All vaccines against COVID-19 have been available in Tunisia and at the Military Hospital of Tunis, but the number of people vaccinated has remained below the expected figure.

At the Military Hospital of Tunis, we decided to make vaccination against COVID-19 compulsory for hospital workers. This measure allowed us to vaccinate 93.3% of the staff completely. This measure was taken in addition to the national decision to consider COVID-19 as an occupational disease for health personnel and to cover all side effects attributable to COVID-19 vaccination by the state. Making vaccination compulsory has already been proposed by several authors for the influenza vaccine and for vaccines recommended to certain professional categories, but has rarely been applied [3,4]. This measure should be combined with state coverage of adverse effects of vaccination [5]. Considering the results of the study on the acceptance of the COVID-19 vaccine conducted at the Military Hospital of Tunis and the decisions taken which have allowed to reach a vaccine coverage rate close to 100%, we believe that only mandatory vaccination will allow to reach a vaccine coverage in the general population higher than 70%.
